# Early pregnancy body mass index and spontaneous preterm birth in Northwest Russia: a registry-based study

**DOI:** 10.1186/1471-2393-14-303

**Published:** 2014-09-04

**Authors:** Ekaterina E Sharashova, Erik E Anda, Andrej M Grjibovski

**Affiliations:** Department of Community Medicine, UiT The Arctic University of Norway, N-9037 Tromsø, Norway; International School of Public Health, Northern State Medical University, Troitski 51, office 1252, 163001 Arkhangelsk, Russia; Department of International Public Health, Norwegian Institute of Public Health, Post box 4404, Nydalen, N-0403 Oslo, Norway

**Keywords:** BMI, Obesity, Preterm birth, Russia

## Abstract

**Background:**

International studies on the association between maternal body mass index (BMI) and spontaneous preterm birth (PTB) yield controversial results warranting large studies from other settings. The aim of this article was to study association between maternal early pregnancy BMI and the risk of spontaneous PTB in Murmansk County (MC), Northwest Russia.

**Methods:**

This is a registry-based cohort study. All women with singleton pregnancies registered at antenatal clinics during the first 12 weeks of gestation and who delivered in MC between January, 1^st^ 2006 and December, 31^st^ 2011 comprised the study base (n = 29,709). All women were categorized by BMI into four groups: underweight (<18.5 kg/m^2^), normal (18.5–24.9 kg/m^2^), overweight (25.0–29.9 kg/m^2^), and obese (≥30.0 kg/m^2^). Multivariable logistic regression was used to study associations between maternal BMI and PTB (<37 weeks) and very preterm birth (VPTB) (<32 weeks) adjusted for socio-economic factors, biological and lifestyle characteristics.

**Results:**

The prevalence of underweight, overweight and obesity were 7.1% (95% CI: 6.8-7.4), 18.3% (95% CI: 17.8-18.7) and 7.1% (95% CI: 6.8-7.4), respectively. Altogether, 5.5% (95% CI: 5.3-5.8) of the births were PTB and 0.8% (95% CI: 0.7-0.9) were VPTB. After adjustment, both underweight (OR = 1.25, 95% CI: 1.03-1.50), overweight (OR = 1.10, 95% CI: 0.97-1.26) and obese (OR = 1.31, 95% CI: 1.08-1.57) women were more likely to deliver preterm. VPTB was associated with overweight (OR = 1.47, 95% CI: 1.056-2.03) and obesity (OR = 1.63, 95% CI: 1.02-2.60).

**Conclusion:**

The findings demonstrate a J-shaped association between first trimester maternal BMI and spontaneous PTB and VPTB with increased risk among underweight, overweight and obese women.

## Background

Obesity has become an epidemic in many parts of the world and is currently a considerable public health problem with implications for maternal and child health. About 45% of Russian women are overweight or obese, although the estimates vary between regions [1]. The prevalence of underweight is much lower than the prevalence of overweight and obesity. Less than 4% of Russian women between the ages of 18 and 49 are underweight [2]. Despite such a low prevalence, underweight contributes to adverse pregnancy outcomes including preterm birth (PTB) representing a challenge for both obstetric and neonatal health care [3].

PTB is a frequent and unfavorable pregnancy outcome defined as delivery before 37 completed weeks of gestation. The prevalence of PTB is 11-12.9% worldwide [4, 5] and 6.2% in Europe [5] while Russian studies have reported a prevalence of 5.2- 8.7% [6–8]. PTB is one of the main contributors to neonatal mortality, neonatal morbidity and childhood morbidity and is associated with considerable burden for health care system [9].

Many studies have examined associations between maternal obesity and PTB, often with conflicting results. The prevalence of PTB among overweight and obese women has been shown to be higher [9–11], lower [3, 12, 13], or similar [14] to the prevalence among women of normal weight. Some studies have reported a U-shaped relationship between maternal BMI and PTB with an increased risk of PTB in underweight and obese women [15]. The effect of maternal underweight on obstetric outcomes is unclear. Some researchers [3, 16] have observed increased risk of PTB among underweight women, while others have reported a protective effect of being underweight in relation to PTB [11, 17]. The data from Russia are scarce. Only one internationally published Russian study on the association between maternal weight and PTB was identified [7]. The findings did not reach the level of statistical significance, but it may be due to the relatively small sample size. Inconsistent results on the associations between maternal BMI and PTB warrant further large-scale studies from different countries. Moreover, given insufficient evidence from Russia combined with the fact that both overweight and underweight is relatively common among women of reproductive age in Northwest Russia; this study may have implications for local health care practices as well as further afield.

Using data from the Murmansk County Birth Registry (MCBR) this study investigates associations between maternal BMI in early pregnancy and PTB.

## Methods

The study was conducted in Murmansk County (MC) which is one of the northernmost counties of Russia bordering Norway and Finland. It covers an area of 114,900 square km and had a population of 787,900 on January, 1^st^ 2012, 93% of which resided in urban areas [18].

This is a registry-based cohort study with data from MCBR. Implementation and quality control of the registry has been described in details elsewhere [19]. Briefly, the MCBR was established in 2005 and covers more than 99.5% of all deliveries in the county. MCBR contains maternal, obstetric and perinatal information on births from all 15 maternity hospitals or maternity units in MC from January, 1^st^ 2006. All the information was collected and recorded on the registry form by the trained physicians and midwifes, the forms were sent by courier to the Registry Office in Murmansk, where the data where entered into an Access database by 2 trained persons (double entry). The quality and completeness of the MCBR has been assessed, and was found to be acceptable.

We excluded women with multiple pregnancy as well as those who had induced deliveries or any type of cesarean section (SC) including intrapartum SC. All women with spontaneous singleton deliveries in MC between January, 1^st^ 2006 and December, 31^st^ 2011 were included into the study. The information on date of birth, ethnicity, education, parity, marital status, place of residence, smoking before pregnancy and alcohol abuse during pregnancy (diagnosed by a doctor) come from the mothers’ medical records and the women themselves through interviews conducted by the trained physicians or midwifes. Information on hyperemesis gravidarum, preeclampsia/eclampsia, gestational diabetes and excessive weight gain during index pregnancy, the date of delivery, date of the first day of the last menstruation period and infant sex are derived from obstetric journals and newborn records. Mothers’ weight and height were measured at the first antenatal visit.

The main outcomes of the study were spontaneous PTB and very preterm birth (VPTB). Among those included into the study, all deliveries: with intact membranes or with preterm ruptured membranes, were considered as long as the onset was spontaneous. Those who had spontaneous onset of delivery, but delivered by intrapartum cesarean section were excluded from the study from the beginning. PTB and VPTB were defined as delivery before 37 and 32 completed weeks, respectively. Moderate preterm birth (MPTB) was also examined separately. It was defined as birth before 37, but after 32 completed weeks of gestation. Gestational age was calculated as the difference between date of birth and the first day of the last menstruation and expressed in weeks.

The primary exposure variable was maternal first-trimester BMI which was calculated as mother’s weight at the first antenatal visit in kilograms divided by height in meters squared. Women who had their first visit after 12 completed weeks of gestation were excluded. By BMI, all mothers were categorized into four groups: underweight (<18.5 kg/m^2^), normal weight (18.5–24.9 kg/m^2^), overweight (25.0–29.9 kg/m^2^), and obese (≥30.0 kg/m^2^).

All selected potential confounders were used as categorical variables. Maternal age was divided into 5 groups: <20 years, 20-24 years, 25-29 years, 30-34 years and 35 years or older. By ethnic background, women were categorized into Russian or other. Marital status was either married or unmarried (included cohabiting, single and widowed). Mother’s education was classified as incomplete secondary or less (9 years of schooling or less), secondary (10-11 years of schooling), vocational, higher or unknown. By parity, women were divided into nulliparous (with no previous deliveries), with at least one previous delivery, and multiparous (with two or more previous deliveries). Residence was dichotomized into urban and rural. Smoking before pregnancy and alcohol abuse during pregnancy were treated as dichotomous variables. Diabetes (Type 1, Type 2 and gestational diabetes), hyperemesis gravidarum, preeclampsia/eclampsia, excessive weight gain during pregnancy (O26.0 according to ICD 10) and infant sex (male/female) were also treated as binary variables.

Categorical variables were analyzed using Pearson’s chi-squared tests. Binary logistic regression was used to study associations between PTB, VPTB, and MPTB and categories of maternal BMI with and without adjustment for potential confounders. Normal BMI was used as a reference category. Crude and adjusted odds ratios (OR) with 95% confidence intervals (CI) were calculated. Multivariable analyses with each of the dependent variables were divided in two sets: first without and then with the pregnancy complications mentioned above. All analyses were performed using SPSS for Windows, version 19 (SPSS Inc., Chicago Il, USA).

MCBR was established with approval of the Regional Health Administration of the Murmansk Oblast and the Ethical Committee of Gynecology – Obstetrician Association Group. The present study was approved by International School of Public Health, Northern State Medical University (NSMU), Arkhangelsk, Russia, by the Ethical Committee of NSMU, Arkhangelsk, Russia and the Regional Committees for medical and Health Research Ethics (REC-North), Tromsø, Norway.

## Results

**Figure 1:**
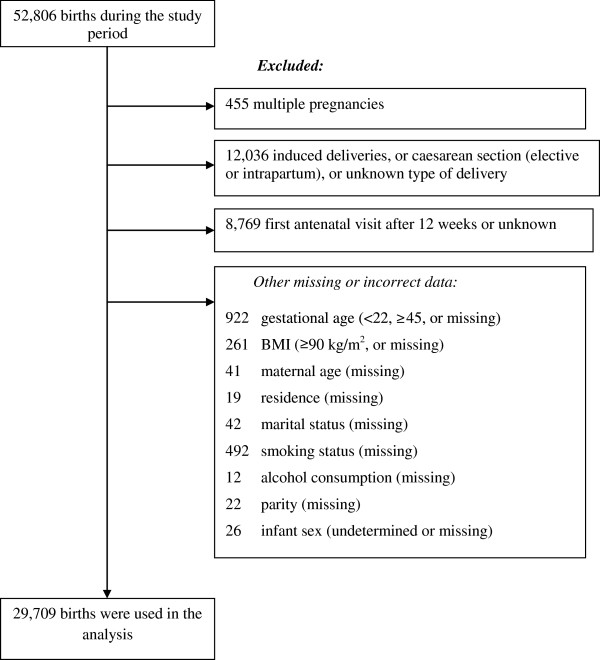
**Number of births during the study period and exclusions.**

A total of 52,806 births were registered in MC from January 1st 2006 to December 31st 2011. Sampling details, missing data and implausible values are presented in Figure 1. Of the 29,709 women who comprised the final sample, 7.1% (95% CI: 6.8-7.4) were underweight, 67.6% (95% CI: 67.1-68.1) were of normal weight, 18.3% (95% CI: 17.8-18.7) were overweight, and 7.1% (95% CI: 6.8-7.4) were obese.

Maternal background characteristics across BMI groups are presented in Table 1. Underweight mothers were younger, had lower education and were more often unmarried. Overweight and obese women were more likely to be rural residents, smoked before pregnancy, and had one or more previous deliveries.

**Table 1:** **Characteristics of women with spontaneous singleton births by early pregnancy body mass index, n = 29,709**

Characteristics	Underweight	Normal	Overweight	Obese	p ^*^
	n = 2,100	n = 20,081	n = 5,427	n = 2,101	
Age at delivery, mean (SD)	24.2 (4.1)	26.4 (4.8)	28.4 (5.2)	29.4 (5.0)	<0.001
Age at delivery, n (%)					<0.001
≤ 19	239 (11.4)	1,257 (6.3)	167 (3.1)	29 (1.4)	
20-24	965 (46.0)	6,510 (32.4)	1,230 (22.7)	345 (16.4)	
25-29	668 (31.8)	7,214 (35.9)	1,812 (33.4)	703 (33.5)	
30-34	191 (9.1)	3,874 (19.3)	1,485 (27.4)	670 (31.9)	
≥35	37 (1.8)	1,226 (6.1)	733 (13.5)	354 (16.8)	
Ethnicity, n (%)					0.039
Russian	1,978 (94.2)	18,957 (94.4)	5,067 (93.4)	1,979 (94.2)	
Other	122 (5.8)	1,124 (5.6)	360 (6.6)	122 (5.8)	
Education, n (%)					<0.001
Incomp. secondary or less	81 (3.9)	448 (2.2)	119 (2.2)	47 (2.2)	
Secondary	745 (35.5)	5,700 (28.4)	1,450 (26.7)	582 (27.7)	
Vocational	624 (29.7)	6,287 (31.3)	1,998 (36.8)	769 (36.6)	
Higher	634 (30.2)	7,516 (37.4)	1,832 (33.8)	690 (32.8)	
Unknown	16 (0.8)	130 (0.6)	28 (0.5)	13 (0.6)	
Marital status, n (%)					<0.001
Married	1,540 (73.3)	15,334 (76.4)	4,038 (79.4)	1,706 (81.2)	
Unmarried	560 (26.7)	4,747 (23.6)	1,119 (20.6)	395 (18.8)	
Residents, n (%)					<0.001
Urban	1,671 (79.6)	16,160 (80.5)	4,276 (78.8)	1,587 (75.5)	
Rural	429 (20.4)	3,921 (19.5)	1,151 (21.2)	514 (24.5)	
Smoking before pregnancy, n (%)					<0.001
Yes	490 (23.3)	4,345 (21.6)	1,269 (23.4)	536 (25.5)	
No	1,610 (76.7)	15,736 (78.4)	4,158 (76.6)	1,565 (74.5)	
Alcohol abuse during pregnancy, n (%)					0.261^†^
Yes	7 (0.3)	31 (0.2)	9 (0.2)	5 (0.2)	
No	2,093 (99.7)	20,050 (99.8)	5,418 (99.8)	2,096 (99.8)	
Parity, n (%)					<0.001
0	1,536 (73.1)	12,053 (60.0)	2,350 (43.3)	725 (34.5)	
1	501 (23.9)	7,027 (35.0)	2,540 (46.8)	1,088 (51.8)	
2+	63 (3.0)	1,001 (5.0)	537 (9.9)	288 (13.7)	
Diabetes mellitus (types 1 or 2 or gest.), n (%)					0.002^†^
Yes	0 (0.0)	27 (0.1)	8 (0.1)	9 (0.4)	
No	2,100 (100.0)	20,054 (99.9)	5,419 (99.9)	2,092 (99.6)	
Hyperemesis gravidarum, n (%)					<0.001
Yes	62 (3.0)	1,223 (6.1)	631 (11.6)	377 (17.9)	
No	2,038 (97.0)	18,858 (93.9)	4,796 (88.4)	1,724 (82.1)	
Preeclampsia/eclampsia, n (%)					0.003^†^
Yes	1 (<0.1)	20 (0.1)	6 (0.1)	8 (0.4)	
No	2,099 (100.0)	20,061 (99.9)	5,421 (99.9)	2,093 (99.6)	
Excessive weight gain during pregnancy, n (%)					<0.001
Yes	98 (4.7)	1,201 (6.0)	387 (7.1)	137 (6.5)	
No	2,002 (95.3)	18,880 (94.0)	5,040 (92.9)	1,964 (93.5)	

^*^Calculated using analysis of variances (for age as an interval variable), or chi-squared test (other variables).

^†^25% of cells in the cross table have expected count less than 5.

The overall proportions of PTB and VPTB were 5.5% (95% CI: 5.3-5.8) and 0.8% (95% CI: 0.7-0.9), respectively. The prevalence of PTB varied between 5.2% in women with normal weight and 6.9% in obese women. The proportion of VPTB varied between 0.7% among women with normal weight and 1.0% among overweight and obese women (Table 2).

**Table 2:** **Associations between body mass index, preterm, moderate preterm and very preterm birth among 29,709 women with spontaneous singleton births**

Response variables	n	% (95% CI)	Crude OR (95% CI)	p	Adjusted OR (95% CI) ^‡^	p	Adjusted OR (95% CI) ^§^	p
*Preterm birth*	1,645	5.5 (5.3-5.8)						
Underweight	138	6.6 (5.6-7.7)	1.28 (1.06-1.54)	0.009	1.26 (1.04-1.51)	0.017	1.25 (1.03-1.50)	0.021
Normal	1,048	5.2 (4.9-5.5)	1	-	1	-	1	-
Overweight	314	5.8 (5.2-6.4)	1.12 (0.98-1.27)	0.099	1.09 (0.95-1.24)	0.215	1.10 (0.97-1.26)	0.152
Obese	145	6.9 (5.9-8.1)	1.35 (1.13-1.61)	0.001	1.29 (1.08-1.56)	0.006	1.31 (1.08-1.57)	0.005
*Very preterm birth* ^*^	234	0.8 (0.7-0.9)						
Underweight	19	0.9 (0.6-1.4)	1.30 (0.80-2.10)	0.285	1.30 (0.80-2.11)	0.292	1.26 (0.77-2.04)	0.356
Normal	140	0.7 (0.6-0.8)	1	-	1	-	1	-
Overweight	53	1.0 (0.8-1.3)	1.41 (1.02-1.93)	0.036	1.39 (1.01-1.92)	0.045	1.47 (1.06-2.03)	0.021
Obese	22	1.0 (0.7-1.6)	1.51 (0.96-2.37)	0.075	1.49 (0.94-2.37)	0.091	1.63 (1.02-2.60)	0.039
*Moderate preterm birth* ^*†*^	1,411	4.8 (4.6-5.0)						
Underweight	119	5.7 (4.8-6.8)	1.27 (1.04-1.55)	0.017	1.24 (1.02-1.52)	0.032	1.24 (1.02-1.51)	0.035
Normal	908	4.6 (4.3-4.9)	1	-	1	-	1	-
Overweight	261	4.9 (4.3-5.5)	1.07 (0.93-1.23)	0.347	1.04 (0.90-1.20)	0.591	1.05 (0.91-1.21)	0.524
Obese	123	5.9 (5.0-7.0)	1.32 (1.09-1.60)	0.005	1.26 (1.03-1.54)	0.022	1.26 (1.03-1.54)	0.026

^*^Compared to moderate preterm and term births.

^†^compared to term births (total n = 29,475).

^‡^Adjusted for marital status, education, place of residence, mothers’ age, ethnicity, parity, smoking before pregnancy, alcohol abuse during pregnancy, and infant sex.

^§^Adjusted for all above-mentioned variables and for diabetes mellitus 1 and 2 types and gestational diabetes, hyperemesis gravidarum, preeclampsia/eclampsia and excessive weight gain during pregnancy (O26.0 according to ICD 10).

Table 2 summarizes crude and adjusted associations between maternal BMI and PTB, VPTB, and MPTB. In crude analysis, underweight and obese women had 28% and 35% higher risk of PTB, respectively compared to the normal weight group. Adjustment for both sets of potential confounders changed the observed J-shaped association only marginally. In the final model, underweight, overweight and obese women had, on average, 25%, 10% and 31% higher risk of PTB compared to the normal weight group, although the effect of overweight was not significant. Merging overweight and obese women into one group resulted in an OR of 1.16 (95% CI: 1.03-1.30).

The crude risk of VPTB was 30% higher in underweight women, 41% higher in overweight, and 51% higher in obese women compared to the normal weight group reaffirming a J-shaped association. Again, the adjustments changed the estimates only marginally. In the final model, the risk of VPTB in underweight women was 26%, in overweight – 47% and in obese women – 63% higher compared to the reference group, although the risk for VPTB among underweight women did not reach a level of statistical significance. Merging overweight and obese women into one group resulted in an OR of 1.51 (95% CI: 1.13-2.02).

The association between maternal BMI and MPTB was U-shaped with 24% higher risk of MPTB among both underweight and obese, and comparable risks among overweight women and the normal weight group. Merging overweight and obese women into one group resulted in an OR of 1.10 (95% CI: 0.97-1.26).

## Discussion

This study adds to the increasing body of evidence that underweight, overweight and obesity in early pregnancy may be associated with PTB. Based on the data from MCBR, a J-shaped relationship was found with increased risk of spontaneous PTB and VPTB while a U-shaped association was observed for MPTB.

### Maternal BMI and PTB

This registry-based cohort study shows that the prevalence of overweight and obesity combined among pregnant women with spontaneous singleton births in MC, Northwest Russia was nearly 25.4% and the prevalence of underweight women was 7.1%. Two studies conducted in Severodvinsk, Northwest Russia in 1999 found a prevalence of overweight and obesity among pregnant women combined to be 4.5% for spontaneous births [7, 20] which is less than one-fifth of the prevalence observed in this study. The prevalence of underweight women in Severodvinsk varied from 5.6% [7] to 8.6% [20], which is comparable with the prevalence in this study. The proportion of spontaneous PTB in MC (5.5%) was also comparable to the proportions in Severodvinsk (5.6%).

We found a J-shaped relationship between BMI and the risk of spontaneous PTB. According to the findings of one of the largest recently conducted systematic reviews the risk of spontaneous PTB did not differ between women with different BMI: combined relative risk (RR) for overweight and obese compared to normal weight women is 0.93 (95% CI: 0.85-1.01, 15 studies) [9]. However the authors underline that a large number of studies presented crude data while only a few presented matched or adjusted data. The pooled RR of spontaneous PTB from adjusted or matched data was 2.29, (95% CI: 1.20-4.38), that is even higher compared to our findings. At the same time, according to the data from 7 case-control studies [9] that used maternal BMI as a continuous variable, women with spontaneous PTB had a slightly lower BMI: mean difference is -0.90 (95% CI: -1.77 to -0.02). This may be explained by a negative effect of underweight that was found in our study as well.

Another large study from the United States conducted on 437,403 underweight and normal weight women with singleton births aimed to estimate the risk of PTB and VPTB by severity of low pre-pregnancy BMI [21]. Maternal BMI was measured at the first antenatal visit and gestational age was based on the interval between the last menstrual period and infant birth date. Underweight compared to normal weight women had an increased risk of spontaneous PTB and VPTB: adjusted ORs are 1.44 (95% CI: 1.40-1.48) and 1.50 (95% CI: 1.42-1.59), respectively. These results correspond to ours although the estimates almost twice as higher.

Some studies suggest that maternal adiposity has a protective effect on the risk for PTB. A register-based cohort study [3] found that the risk of VPTB (<33 weeks of gestation) did not increase in the overweight, obese or morbidly obese women, while the risk of PTB (<37 weeks of gestation) was significantly lower among overweight and obese women. Underweight women had a significantly elevated risk of PTB and VPTB. The authors, however, noted that they were unable to differentiate between spontaneous and induced PTB. Maternal BMI was calculated based on weight and height measured during the first 16 weeks of pregnancy rather than at 12 weeks as measured in this study. Thus, maternal BMI could be overestimated. They also had a high proportion of missing BMI data – 37% of the population, which could result in biased findings.

According to the latest systematic review of the literature with meta-analysis overweight and slightly obese (BMI 30.0-34.9 kg/m^2^) compared to normal weight women have a 15% reduction of adjusted risk of spontaneous PTB gestation [22]. We did not find any significant effect of overweight group. The same review [22] found just a few cohort studies on spontaneous MPTB and VPTB, and none of them adjusted for possible confounders. Among the limitations the authors mention high heterogeneity in the risk estimates between the studies that is due to due to differences in participant selection, non-uniform BMI categorization and the lack of uniform definition for spontaneous PTB.

Another systematic review aimed to estimate the accuracy of antenatal maternal anthropometric measurements in predicting the risk spontaneous PTB [4]. The authors concluded that routine anthropometric measurements are not useful in predicting the risk of PTB. The conclusion was based on high heterogeneity in anthropometric measurements and PTB definition. Additionally, none of the studies’ design fulfilled the criteria of good quality. According to the authors, more studies should be taken and a more clinically appropriate reference standard of PTB (e.g. before 32–34 weeks’ gestation) should be applied. We used the most common WHO classification of BMI, and several definitions of PTB.

### Limitations of the study

Several important limitations should be considered when interpreting the results of this study. Initially, it comprised a rather large population (52,806 births from MC during six years). After exclusion of women with multiple pregnancies, medically induced deliveries, late first antenatal visit and women with missing or incorrect data, 29,709 births were finally included in the analysis. According to their demographical and life style characteristics, however, excluded women were just slightly different from the study base.

The ideal time to record the baseline height and weight of a pregnant woman is before she has started to gain weight due to gestation. Given that a woman’s weight measured before the pregnancy is difficult to obtain, (and was not available in the MCBR), the preferred approach would be to have the weight measured early during prenatal care. A recent study showed that there was no significant change in maternal BMI during first trimester [23].

It is also a well-known limitation to measure gestational age based on the last menstrual period as there may be recall errors [24]. However, this would result in non-differential misclassification and bias towards the null.

Maternal characteristics as diabetes, hyperemesis gravidarum, and pre-eclampsia/eclampsia were introduced into the second set of the multivariable model as possible confounders. These diseases are pregnancy complications and also associated with obesity. Thus, there is a possibility that they are intermediate factors and lie along the causal pathway between overweight/obesity and PTB. This possibility raises questions for future studies of whether the BMI – PTB relationship is mediated by unmeasured disease processes, or by endocrine changes in obese women during or prior to pregnancy.

This study did not take into account gestational weight gain which could be a confounder, intermediate factor, or effect modifier. However, the associations of interest were adjusted for excessive weight gain during pregnancy, and there was no significant interaction between excessive weight gain during pregnancy and BMI on the risk of PTB, VPTB and MPTB (not presented in the results).

We had also no opportunity to adjust for the association between maternal BMI and the risk of PTB for fertility treatments and other medication use, history of preterm delivery and/or pregnancy loss, psychological stress, physical activity, income, and some other potential confounders. Some of the information was self-reported, particularly such sensitive item as smoking before pregnancy that could result in underestimation and measurement bias.

One more of the primary inherent limitations in this study is the uncertainty of whether observed associations are causal. Overweight and obese women are thought to have additional fat stores which are part of the normal physiological weight gain during pregnancy. This might possibly diminish the effect of their weight gain on the birth weights of their infants. Also, the association between maternal obesity and PTB is still controversial; there are some possible biological explanations of increased risk of PTB among overweight and obese women. According to a recent study, inflammation or infection related to obesity seems to be part of the causal pathway to increase the risk of spontaneous PTB [25]. Finally, obesity is often associated with abnormal vaginal flora during pregnancy leading to chorioamnionitis and/or premature rupture of membranes that, again, might cause PTB.

## Conclusions

Underweight, overweight, and obesity were all found to be associated with both PTB and VPTB in Northwest Russia. This should be taken into account when planning pregnancy. These high risk categories should be given special attention during pregnancy. The absolute values of the coefficients are comparable to those obtained in larger European studies and can be used in subsequent meta-analyses.

## Abbreviations

BMI: Body mass index
MCBR: The Murmansk County Birth Registry
MPTB: Moderate preterm birth
OR: Odds ratio
PTB: Preterm birth
RR: Relative risk
VPTB: Very preterm birth
